# Are Private Reserves Effective for Jaguar Conservation?

**DOI:** 10.1371/journal.pone.0137541

**Published:** 2015-09-23

**Authors:** Carmina E. Gutiérrez-González, Miguel A. Gómez-Ramírez, Carlos A. López-González, Paul F. Doherty

**Affiliations:** 1 Laboratorio de Zoología, Facultad de Ciencias Naturales, Universidad Autónoma de Querétaro. Santiago de Querétaro, Querétaro, México; 2 Department of Fish, Wildlife and Conservation Biology, Colorado State University, Fort Collins, Colorado, United States of America; Oregon State University, UNITED STATES

## Abstract

We present the first study of density and apparent survival for a jaguar (*Panthera onca*) population in northern Mexico using 13 years of camera trap data from 2000 to 2012. We used the Barker robust design model which combines data from closed sampling periods and resight data between these periods to estimate apparent survival and abundance. We identified 467 jaguar pictures that corresponded to 48 jaguar individuals. We included camera type and field technician as covariates for detection probabilities. We used three covariates to evaluate the effect of reserve on jaguar apparent survival: i) private reserve creation ii) later reserve expansions, and iii) cattle ranches’ conservation activities. We found that the use of digital cameras in addition to film cameras increased detection probability by a factor of 6x compared with the use of only film cameras (p = 0.34 ± 0.05 and p = 0.05 ± 0.02 respectively) in the closed period and more than three times in the open period (R = 0.91 ± 0.08 and R = 0.30 ± 0.13 mixed and film cameras respectively). Our availability estimates showed no temporary emigration and a fidelity probability of 1. Despite an increase of apparent survival probability from 0.47 ± 0.15 to 0.56 ± 0.11 after 2007, no single covariate explained the change in these point estimates. Mean jaguar density was 1.87 ± 0.47 jaguars/100 km^2^. We found that 13 years of jaguar population monitoring with our sampling size were not enough for detecting changes in survival or density. Our results provide a baseline for studies evaluating the effectiveness of protected areas and the inclusion of ranch owners in jaguar conservation programs and long-term population viability.

## Introduction

Jaguar (*Panthera onca*) conservation has been the focus of many studies for more than 20 years and populations are perceived to be declining [1]. The jaguar is a long-lived species that reaches maturity at three years old and presents a low reproductive potential [2], implying that high survival is needed for populations to be viable, and because of that, jaguar requirements for long-term persistence need to be addressed [3]. Yackulic *et al*. [4] found that natural protected areas are benefical for the survival of different felid species if human activities are controlled; however, human activities and conflicts associated with cattle depredation are primary threats for jaguar populations [5–8].

Various strategies have been developed to conserve jaguars, including establishment of government and private reserves and connecting corridors [9]. As an effort to prioritize areas for jaguar conservation, in 2002, Jaguar Conservation Units (JCU) were proposed [10]. Each JCU is intended to encompass a viable jaguar population and allow for its long-term (>100 years) persistence. These units are designated based on qualitative evaluation of habitat availability and habitat connectivity, stable prey base, and reduced level of threat from human activity.

Assessing the effectiveness of protected areas has been hampered because most jaguar studies are short term (e. g., 2–3 months) and have focused primarily on density, home range and dietary preferences (e.g., [11–15]). Jaguar monitoring projects are needed to assess the long-term efficacy of protected areas for species conservation. Estimates of survival, recruitment and dispersal are needed, especially in areas where potential livestock depredation has led to conflict and possible human-caused jaguar mortality [16]. Jaguar survival is poorly understood in Mexico and throughout Central America (e.g., [17–19]). Here we present an approach for long-term data analysis that combines closed and open population information (i.e., the Barker robust design model) to estimate demographic parameters [20]. This model allows parameter estimation over time in areas where jaguar populations have been monitored for several years.

We conducted our study in the Northern Jaguar Reserve in northeastern Sonora, México. This reserve was established in 2003 to aid in jaguar conservation. It is a private reserve without cattle owned and managed by an NGO. We used 13 years of mark-resight data from camera traps surveys in the Northern Jaguar Reserve and surrounding areas to estimate jaguar density and apparent survival. We also evaluated potential effects of reserve establishment and its management strategy (i.e., cattle removal) on jaguar apparent survival. We predicted that survival and density would increase post reserve establishment.

## Methods

### Study area

**Fig 1:**
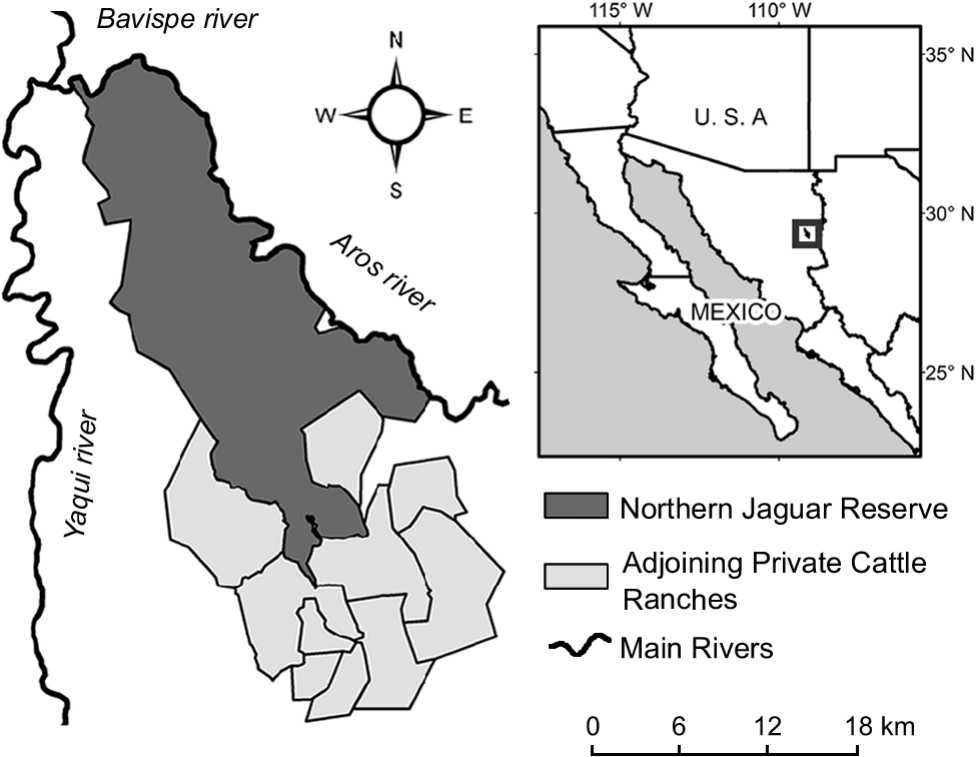
Study area in 2012. Northern Jaguar Reserve and cattle ranches near the reserve. The reserve is private land without cattle. Since 2007, cattle ranches have been enrolled in an agreement for jaguar and other feline protection in the eastern part of Sonora, Mexico.

The study area is located in northeastern Sonora, Mexico, between 29° 32.4 'N—109° 14.4' W and 29° 12 'N—108° 58.8' W. It is comprised of the Northern Jaguar Reserve and cattle ranches adjacent to the reserve. The reserve is nested within a JCU proposed by Sanderson *et al*. [10] and is intended to serve as core habitat for a portion of the northernmost jaguar population in North America [10, 21]. In this area, 1.04 ± 0.04 jaguars per 100 km^2^ were reported for 2009 [17]. The area is composed of a series of sierras, with altitudes ranging from 370 to 1600 m, and it is naturally limited on the north by the junction of two major rivers (Fig 1). Because of its isolated location, it is an area with few significant human impacts. The climate is mostly semiarid with annual precipitation between 400 and 800 mm [22]. The annual average temperature is over 18°C. Vegetation types include desert scrub and thornscrub mostly with a tropical affinity [23]; dominant shrub species in the area include *Lysiloma watsonii*, *Prosopis velutina*, *Vachelia campechiana* and *Jatropha cordata*. The presence of tropical deciduous forest in some canyons and shaded hillsides is represented by *Bursera* spp. and *Ipomoea arborescens*. Oaks (*Quercus* spp.) can be found at elevations > 1000 m and in moist shaded canyons. Dominant species in river corridors include *Prosopis velutina*, *Sabal uresana*, *Brahea brandegeei*, *Havardia mexicana*, *Salix bonplandiana*, *Baccharis salicifolia* and *Ambrosia ambrosioides*. Natural grasslands and human-induced grasslands (e.g., *Penisetum ciliare*) and *Dodonaea viscosa* appear mixed with oak woodlands or thornscrub.

**Fig 2:**
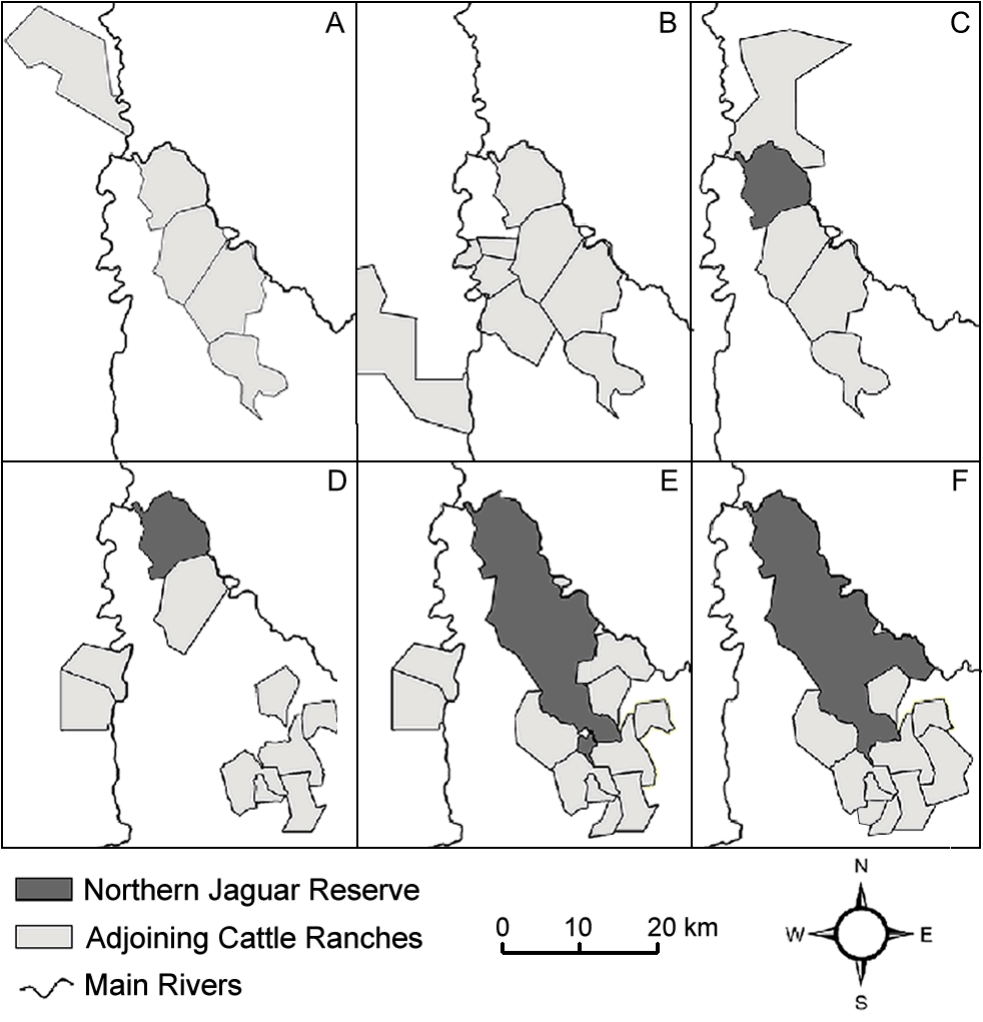
Changes in study area from 2000 to 2012. The extension of the study area is due to ranchers’ permission for monitoring in the area and camera availability. Darker areas correspond to land purchased for the reserve creation and light polygons correspond to cattle ranches. A) study area in 2000, B) study area from 2001 to 2002, C) study area from 2003 to 2006, D) study area from 2007, E) study area from 2008 to 2010, F) study area from 2010 to 2012.

When designated in 2003, the Norther Jaguar Preserve was ~4000 ha, and at that time cattle were removed from this area. The reserve expanded by ~14,000 ha in 2008 and by ~2,000 ha in 2011, for a new total reserve size of ~20,000 ha. All expansions consisted of land purchases contiguous with the original reserve and the primary conservation action was to remove cattle. In 2007, adjacent cattle ranches covering ~13,000 ha signed a conservation agreement to ban wildlife hunting on their lands. This agreement was considered a second conservation action. Additional ranches were included to the conservation agreement action banning hunting from 2007 to 2012. After 2007, we considered the reserve and cattle ranches as a single conservation area for a total study area of ~33,000 ha (Fig 2).

### Field work

We used camera traps and their detections as captures and recaptures of individuals. Different field technicians have monitored the area since 1999. For each ranch, the number, model and type of cameras that we used varied each year. Only Camtrakker 35mm film cameras (Camtrakker, Watkinsville, GA, USA) were used from 2000 to 2008. In 2008, we included Wildview Xtreme 5.0 digital cameras (Wildview, Grand Prairie, TX, USA) in the study. In 2009, we included Cuddeback Capture and Attack (Non Typical Inc., Green Bay, WI, USA) digital cameras. Film cameras were removed from the study in 2010 leaving only digital cameras in the study area. Regardless of the type and model, we set all cameras to have five minutes between capture events and recorded photos 24 hours a day. We checked cameras monthly, and batteries and memory cards were changed in each camera as necessary. We did not use bait or lures to attract animals during the study.

When camera availability allowed, we placed cameras in pairs in order to photograph both sides of an individual [15, 24]. Camera traps were separated by ≥ 1 km and placed in streams, roads, and trails used by wildlife [25]; we changed locations of cameras throughout the study to maximize detections.

### Data analysis

We archived all jaguar pictures taken with camera traps between January 2000 and September 2012, and later we identified individual jaguars by spot patterns [24]. We determined sex of the individual when possible. We eliminated from the analysis pictures that precluded identification through spot patterns.

We built a detection history of each individual with records from 2000 to 2012. We eliminated one juvenile record because juveniles can present different survival and recapture probabilities [26]. We did not have dead recovery information for the encounter histories [27]. Without dead information recoveries, and searching a larger area, it is impossible to know if individuals leave the study area or die [28]. Thus, we viewed the survival parameter as apparent survival.

We used the Barker robust design model for data analysis [20]. This model requires a robust design component [29] with a detection history composed of secondary and primary sampling occasions and includes auxiliary observations of individuals between primary occasions (Barker model [30]). This combined model can improve survival estimates in comparison with the robust design estimates or the Barker model alone [20]. For a complete description of the Barker robust design model see Kendall *et al*. [20]. Within each primary period (year), we considered each of 4 months (Feb, Mar, Apr, May) as secondary sampling periods (closed periods). We tested the closure assumption during this period by comparing a closed population model (entry probability = 0 and survival probability = 1) versus an open model (with entry and survival probabilities estimated) [31]. We coded a 1 if an individual was photographed at least once in a month, and a 0 in months when the individual was not detected. Resighting data during the rest of the year (open period) were included after the fourth sampling period (May).

The Barker robust design model includes nine parameters. Because an “all models approach” would give us more than 68,000 models to build, we used an ad hoc, step down approach for model construction [26, 32]. We started with a full-time dependent model in all parameters, but dead encounters parameters (r and Rʹ) were fixed to zero [33]. See S1 Table for a full description of our model structures and hypotheses. In brief, we first investigated capture (p) and recapture (c) (detection) probabilities, addressing possible effects of individual heterogeneity, trap-response behavior, and time-related variation in detection probabilities [34–36], including camera type and field technician. These effects were also investigated for the probability of live detection between primary periods (R).

After we identified a best supported model for p, c and R, we tested if individual availability in primary periods (aʹ and aʺ), was dependent on the availability in the previous period (markovian movement) or not (random movement). We also included a no movement model with aʹ = 0 and aʺ = 1 [28]. Availability parameters are considered the complement of temporary emigration [20] and allow the researcher to determine the probability that an individual is in or out the study area for the sampling period. Finally, we modeled fidelity (F) and apparent survival (phi) probabilities with and without time dependence, with reserve and conservation effects (S1 Table). We obtained an estimate of abundance (N) as derived parameter. Additionally, we tested for transience by considering different apparent survival probabilities for new individuals and recaptured individuals [26, 37]. All analyses were performed using program MARK ver 7.1 and the Barker robust design model [38].

From all the jaguar pictures, we selected two males who had the most records and calculated a mean of the maximum distance of movement (MMDM) [39] to estimate the effective sampling area [40]. MMDM was estimated as the mean of the sum of all distances between two capture sites (camera locations) [39]. We used the mean obtained as a radius to calculate a circular buffer around camera locations to give an estimate of the sampling area by year [24, 26]. We calculated the effective sampling area annually because the number and location of cameras changed [26, 41]. With the change in sampling area (due to camera availability and ranchers’ permission for monitoring), the number of individuals exposed to the detection varied by year. In order to compare between years, we divided abundance estimates by the estimated sampling area to obtain a relative density estimate per year [26, 31]. We consider that our estimate of relative density is correlated with true density.

## Results

**Fig 3:**
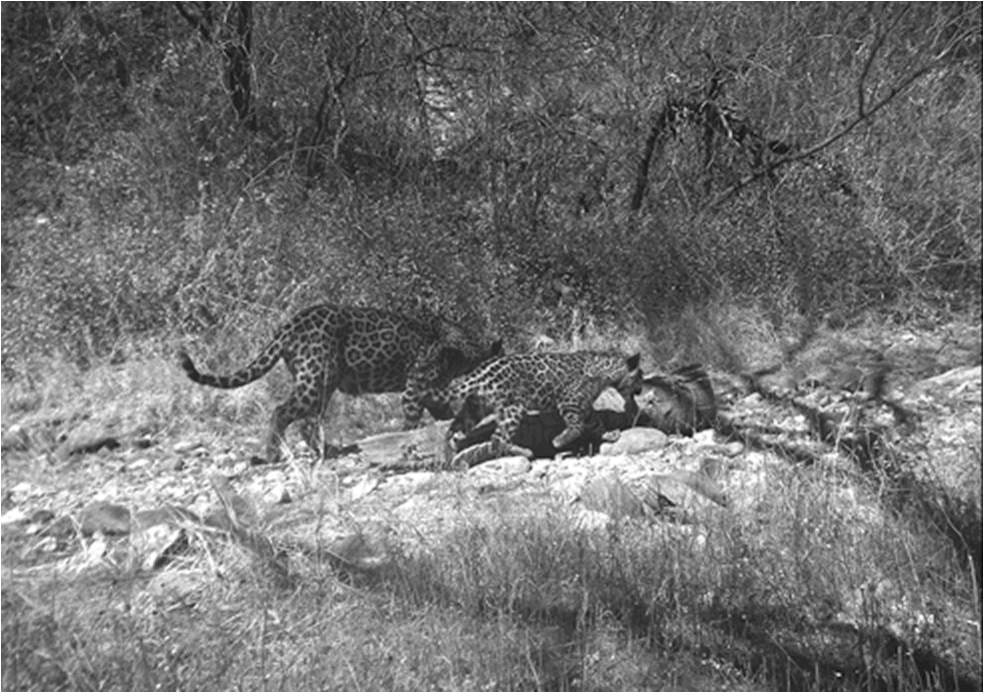
Female jaguar and her cub photographed in February 2011 at the reserve. The female (JH-12) stayed in the area for almost two years. The cub (JNI-18) was never detected again. Juveniles were not included in the study because of their low capture probability. Photo credit: Northern Jaguar Project/Naturalia A.C.

We obtained 467 jaguar pictures that corresponded to 48 individuals. Because of the requirements for Barker robust design detection histories which are conditioned to the first capture in the closed period [20], we did not include 13 individuals that were detected only once during the open period and one juvenile detected in 2011 (Fig 3), leaving 34 individuals for the analysis, 12 females, 14 males, and 8 individuals whose sex could not be determined. The proportion of males and females was not different (z test, p = 0.61) for our data set, and we did not consider sex in the analysis. The average residency for a female in the study area was 2.5 ± 0.50 years and 1.7 ± 0.25 years for a male and the maximum residency recorded for this study was 6.1 and 3.5 years for a female and a male respectively.

We built 30 models to test our hypotheses. The best supported model included an effect of reserve creation for apparent survival (phi), and the mixture of digital cameras and film cameras for the detections on the primary (R) and secondary periods (p, c). Availability parameters (aʹ, aʺ), as well as fidelity (F) remained constant (see S1 Table for a full explanation of notation and models), but because of model uncertainty (Table 1), we decided to calculate model average estimates for apparent survival, as well as detection probabilities and the abundance estimates [42].

**Table 1:** List of the eight best supported models for a jaguar population in northern Mexico.

Model	AICc	Δ AICc	AICc Weights	Model Likelihood	Number of Parameters
phi_(RESERVE EST.)_ R_(FILM VS MIXED)_ p = c_(FILM VS MIXED)_	343.28	0.00	0.36	1.00	6
phi_(RESERVE+RANCHES)_ R_(FILM VS MIXED)_ p = c_(FILM VS MIXED)_	343.44	0.16	0.33	0.92	6
phi_(RESERVE EXP.)_ R_(FILM VS MIXED)_ p = c_(FILM VS MIXED)_	345.46	2.17	0.12	0.34	7
phi_(T/RESERVE EST.)_ R_(FILM VS MIXED)_ p = c_(FILM VS MIXED)_	346.35	3.07	0.08	0.22	8
phi_(T/RESERVE + RANCHES)_ R_(FILM VS MIXED)_ p = c_(FILM VS MIXED)_	346.80	3.52	0.06	0.17	8
phi_(RESERVE STEPS)_ R_(FILM VS MIXED)_ p = c_(FILM VS MIXED)_	348.01	4.73	0.03	0.09	8
phi_(T/RESERVE EXP.)_ R_(FILM VS MIXED)_ p = c_(FILM VS MIXED)_	351.60	8.32	0.01	0.02	10
phi_(TIME)_ R_(FILM VS MIXED)_ p = c_(FILM VS MIXED)_	366.89	23.61	<0.01	0.00	10

Phi-apparent survival probability, R-resight probability in the open period, p-capture probability in the closed period, c-recapture probability in the closed period. RESERVE + RANCHES refers to the model tested for the inclusion of the adjoining ranches to the conservation agreement, RESERVE EST. was the model that tested for the first ranch purchase in 2003, RESERVE EXP. tested the first and second ranch purchase (2003 and 2008), RESERVE STEPS tested each ranch purchase (2003, 2008, 2010), T/ tested the first capture in a closed period as a different class (transient model), TIME tested a time effect, FILM VS MIXED tested the use of different camera model (film camera versus a mixture of film and digital cameras) in the capture and recapture probability.

We found no evidence for trap response or heterogeneity in detection probabilities, but these parameters were influenced by the camera model used in different years. The use of digital cameras increased detection probability during the closed periods 6.8 times (p = 0.05 ± 0.02 with film cameras *vs* p = 0.34 ± 0.05 with mixed cameras). The same effect was observed with the resight probability (R) in open periods where the probability increased 3 times after 2008 (R = 0.3 ± 0.13 with film cameras and R = 0.91 ± 0.08 with mixed cameras).

Availability (aʺ), which is the complement of temporary emigration for primary periods [20], was constant across years in our model (aʺ = 1). The best supported model showed a fidelity probability of 1. Apparent survival probability increased from 0.47 ± 0.15 at the begining of the study to 0.56 ± 0.11 in 2012 (Table 2).

**Table 2:** Apparent survival, abundance and density estimates obtained from jaguar population data from 2000 to 2012 in northern Mexico.

Year	Phi ± SE	N ± SE	D ± SE
2000	0.47 **±** 0.15	10.45 **±** 3.15	1.03 **±** 0.31
2001	0.48 ± 0.16	6.81 **±** 3.18	0.81 **±** 0.38
2002	0.48 ± 0.16	5.89 **±** 3.26	0.77 **±** 0.42
2003	0.48 ± 0.16	7.72 **±** 3.14	1.79 **±** 0.73
2004	0.55 ± 0.12	5.04 **±** 3.36	1.05 **±** 0.70
2005	0.55 ± 0.12	6.86 **±** 3.58	1.25 **±** 0.65
2006	0.55 ± 0.12	6.86 **±** 3.58	2.25 **±** 1.17
2007	0.56 ± 0.12	2.13 **±** 0.58	0.22 **±** 0.06
2008	0.57 ± 0.11	0.31 **±** 0.68	0.03 **±** 0.07
2009	0.56 ± 0.11	5.89 **±** 1.26	0.58 **±** 0.12
2010	0.56 ± 0.11	4.06 **±** 0.92	0.40 **±** 0.09
2011	0.56 ± 0.11	7.72 **±** 1.73	0.73 **±** 0.16
2012	0.56 ± 0.11	5.90 **±** 1.28	0.64 **±** 0.14

Apparent survival (phi) as well as abundance (N) estimates were model averaged due to model uncertainty. Density (D) = abundance (N)/sampling area, expressed as number of individuals per 100 km^2^; SE = standard error.

## Discussion

Our study is the first long-term assessment of jaguar apparent survival and abundance estimation. Due to low detection probabilities, low jaguar numbers in the closed periods, and difficulty in classifying the sex of some individuals, it was not possible for us to include the sex as a categorical variable. However we recognize the possibility that males and females can exhibit differential survival [43] and movement patterns. We also acknowledge that abundance estimates can be negatively biased if sex is not included in the analysis [44].

Our data did not strongly support our primary hypothesis that apparent survival of jaguars would increase after the first land purchase and cattle removal in 2003. We found an additive effect correlated with the incorporation of neighboring cattle ranches with a wildlife conservation agreement in 2007 (Table 2). While the survival estimates increased after the first 4 years of the study, this change was not statistically significant. However, the increase may be biologically meaningful, if increase continues. Thus, we cannot conclude that a measurable change in survival is the result of the conservation strategy. Because our study is unreplicated, we also recognize the possibility that our results can be confounded by alternative temporal and spatial effects, (e.g. years with atypical weather, small sampling area at the begining of the study). However, our case study provides a benchmark to monitor populations, but conservation effects must be inferred with caution.

The conservation agreement action in the cattle ranches (hunting ban) may provide benefits for long-term jaguar conservation without the need for large land purchases. Our results showed an increase from 0.47 to 0.56 in the apparent survival probability after the conservation agreement in the cattle ranches. However, the differences in survival estimates between years were not measurably different (Table 2). Because of the long natural life span of jaguars [21], it is possible that a period of 10 years of camera monitoring after the Northern Jaguar Reserve creation is not enough time to detect jaguar population changes. There is no other long-term jaguar survival study to compare our results, however, Lebreton *et al*. [45] suggest that a 20 year monitoring period is desirable for evaluating survival using mark-resight or recapture methods. We strongly suggest continuation of jaguar monitoring in the Northern Jaguar Reserve area, and that similar long-term monitoring methods be adopted in other priority areas for jaguars to increase the ability to detect population changes.

We calculated annual density estimates, because of variation in camera availability and permissions to access different areas across years (Table 2). Density estimates appeared to decline after 2007 which may have been a result of increases in the sampling area (Fig 2). Maffei and Noss [46] suggest that small sampling areas may overestimate densities by generating buffers that are smaller than animal home range sizes. If sampling area increases, density estimates can become more precise as buffer calculations for effective sampling area begin to approximate the home range of the species.

Additionally, the precision of our density estimate was insufficient to detect annual changes. Because of the inherently low density nature of the species, we are cautious about over-interpreting reductions in density. Additional years of data and better quality digital cameras will allow trends in density to be more precisely estimated and modeled. If density remains essentially unchanged, a plausible explanation is that jaguars may be at carrying capacity in the area [19]. This speculative effect needs to be explored with further research on the relationship between prey abundance and predator density [47].

If we assume that this population is subject to illegal hunting beyond the reserve and ranches with conservation agreements, we recommend extending conservation efforts to areas without agreements. If the population is at carrying capacity and residency and apparent survival increase, new jaguar individuals will need additional areas in which to disperse and thrive [19]. Even with high hunting mortality outside of protected areas, jaguar population persistence may be possible with a few individuals arriving to private reserves through corridors [9].

Transient jaguars that arrive to the study area may not be detected more than once, while other jaguars may disperse into the area and become established as residents. The format for the encounter history that we used requires at least one detection in the closed period before we could document a resight in the open period [48], consequently we ignored 13 individuals that were detected during the open periods and we considered them transients [49] or individuals that may have died before a detection was documented during the closed period. These individuals were not detected again in the study [26, 50]. Sometimes such individuals are considered as part of the population in models [26]. Other authors suggest that individuals that are captured only once in the study are transients and should not be considered in the analysis or should be considered as a different group [26, 51]. Accounting for potential transients can improve accuracy and precision of estimates [50]. We recommend including transience models if detection history allows their inclusion.

We found that variation in detection probabilities among camera types resulted in positively biased estimates of abundance parameters from 2000 to 2006. The same effect was found by other authors [52–54]. We also included field technician as a covariate to account for heterogeneity among observers, but we found that models that included field technicians as a covariate were not well supported in our analysis compared with models that considered camera type (Table 1). Even with the inclusion of camera type as a covariate, we found an overestimation of abundance in years where film cameras were used, and less variation in estimates from the monitoring with digital cameras. The effects of camera type on detection are often ignored [53] therefore, we recommend including camera type effects in future analyses to increase precision and lessen bias in abundance estimates.

We speculated that our resighting probabilities between primary periods (R) are the result of our high monthly sampling effort in the open period. However, considering the cost and logistics of jaguar monitoring projects, we recommend reducing the sampling effort in the open period to a couple of months. This level of effort ensures a detection probability equal to the one in the closed period. The sampling effort needed in the open period could be investigated *a priori* with simulations [37].

Illegal shooting of jaguars still occurs in Mexico, and this factor makes obtaining dead recovery reports difficult. If dead recovery data were available, they should be included in the model for more accurate availability and fidelity estimates and true survival could be estimated [37]. With our mark-resight methods we were not able to differentiate between death or permanent emigration. We considered as residents those individuals that were documented ≥ 2 years (n = 14); after 2 years, these individuals were assumed to be a minimum age of three years old and they have already established their territory [21]. As residents, these individuals are less likely to continue moving and they should continue in the area, even if they are not detected in one sampling period. If these individuals are not detected again though, there is a high likelihood the animal is dead.

Private ranches with proactive conservation actions can provide habitat for jaguars and potential corridors for their dispersal [55]. Agreements with private landowners in priority areas for jaguar conservation may assist with jaguar conservation by functionally adding to reserve size within JCUs, ameliorating edge effects [56], or providing corridors within critical areas [9]. However success in establishing and maintaining effective agreements depends on building relationships between ranchers and authorities.

Overall our study evaluates the efficacy of jaguar reserve and conservation agreements. Our study can also serve as a baseline for future investigations with the objective to evaluate reserve effectiveness for long-term jaguar conservation and we recommend the publication of additional long-term analysis as well as comparative studies between different jaguar populations.

## Supporting Information

S1 Table List of parameters, description and hypotheses tested in the analyisis. ^1^Taken from [13]. ^2^Apparent survival (phi) is used in this study because of the lack of dead recovery information. ^3^Model structure taken from [27]. ^4^Model structure taken from [23] considering that availability paramenters (aʹ and aʺ) are the complement to temporary emigration (ɣʹ and ɣʺ).
(DOC)

S2 Table Jaguar information from 2000 to 2004 used for the capture history. The code for each jaguar represents if it is a male (JM), female (JF) or unknown sex (NI). If an individual jaguar was captured in the closed period (Feb-May) it was coded as a 1, if it was dedected in the open period, it was coded as 2.
(DOC)
