# Supplementary material for: Are Private Reserves Effective for Jaguar Conservation?
Source: PLoS One. 2015 Sep 23;10(9):e0137541. doi: 10.1371/journal.pone.0137541 (PMC4580466; doi:10.1371/journal.pone.0137541)
Supplement: S1 Table — 1Taken from [13]. 2Apparent survival (phi) is used in this study because of the lack of dead recovery information. 3Model structure taken from [27]. 4Model structure taken from [23] considering that availability paramenters (aʹ and aʺ) are the complement to temporary emigration (ɣʹ and ɣʺ). (DOC) [file pone.0137541.s001.doc]

| **Parameter** | **Description^1^** | **Name in models** | **Explanation** | **Hypothesis** |
| --- | --- | --- | --- | --- |
| phi^2^ | Probability that individual *i* in primary sampling period *t* survives to period *t + 1.* | phi(t) | Time variyng | phi (t) |
|  |  | phi(_RESERVE EST)_ | Before Reserve  Reserve Establishment | phi (2000-2003)  phi (2004-2012) |
|  |  | phi(_RESERVE EXP_) | Before Reserve Est. | phi (2000-2003) |
|  |  |  | Reserve Est. | phi (2004-2008) |
|  |  |  | Reserve Expansion | phi (2009-2012) |
|  |  | phi(_RESERVE STEPS_ ) | Before Reserve | phi (2000-2003) |
|  |  |  |  |  |
|  |  |  | Reserve Establishment | phi (2004-2008) |
|  |  |  |  |  |
|  |  |  | 1st Reserve Expansion | phi (2009-2010) |
|  |  |  | 2nd Reserve Expansion | phi (2011-2012) |
|  |  | phi(_RESERVE + RANCHES_) | Before ranches agreement | phi (2000-2006) |
|  |  |  |  |  |
|  |  |  | After ranches agreement | phi (2007-2012) |
| p, c | p=Probability that individual *i* is detected in sample *j* in primary period *t*, given that it is alive, in the population, and available for detection. c=Probability that individual i is detected in sample j in primary period t, given that it was detected in the study areapreviously in primary period t. | M_0_^2^ | Constant | p(.)=c(.) |
|  |  | M_t_^2^ | Time variyng | p(t)=c(t) |
|  |  | M_b_^2^ | Behavioral response | p(.), c(.) |
|  |  | M_h_^2^ | Heterogeneity | p_a_(.)=c_a_(.),π |
|  |  | p=c(_CAM TYPE_) | Film cameras | p(2000–2007) |
|  |  |  | Mixed cameras | p(2008–2009) |
|  |  |  | Digital cameras | p(2010–2012) |
|  |  | p=c(_FILM_ _vs_ _MIXED_) | Only film cameras | p(2000–2007) |
|  |  |  | Mixed cameras | p(2008–2012) |
|  |  |  |  |  |
|  |  | p=c(_MIXED vs DIG_) | Mixed cameras | p(2000–2009) |
|  |  |  | Only digital cameras | p(2010–2012) |
|  |  |  |  |  |
|  |  | p= c(_TECH_) | Different field technician | p(2000–2003) |
|  |  |  |  | p(2004–2005) |
|  |  |  |  | p(2006–2007) |
|  |  |  |  | p(2008) |
|  |  |  |  | p(2009–2011) |
|  |  |  |  | p(2012) |
| a′′ | Probability individual *i* is available for detection in primary period *t + 1*, given it was available in period *t*, and survived and remained faithful to the population from period *t* to *t + 1.* | Markovian^3^ | Time variyng | a"(t) |
|  |  | Random^3^ | Constant | a"(.)=a'(.) |
|  |  | No movement^3^ | Fixed to 1 | a"(1) |
| a′ | Probability individual *i* is available for detection in primary period *t + 1*, given it was unavailable in period *t*, and survived and remained faithful to the population from *t* to *t + 1*. | Markovian^3^ | Time variyng | a' (t) |
|  |  | No movement^3^ | Fixed to 0 |  |
| F | Probability that individual *i* is part of the study population in primary sampling period *t + 1*, given that it is part of the population in period *t*, and survives from period *t* to *t + 1* | Same as phi | | |
|  |  |  |  |  |
|  |  |  |  |  |
|  |  |  |  |  |
|  |  |  |  |  |
|  |  |  |  |  |
|  |  |  |  |  |
| R | Probability that individual *i* is detected alive and reported between primary periods *t* and *t* + *1*, given it survives to period *t* + 1 | Same as p and c with covariables | | |
| N | The size of the subset of the population that is available in the study area during primary period *t.* | Derived parameter | | |
