# Supplementary material for: Are Private Reserves Effective for Jaguar Conservation?
Source: PLoS One. 2015 Sep 23;10(9):e0137541. doi: 10.1371/journal.pone.0137541 (PMC4580466; doi:10.1371/journal.pone.0137541)
Supplement: S2 Table — The code for each jaguar represents if it is a male (JM), female (JF) or unknown sex (NI). If an individual jaguar was captured in the closed period (Feb-May) it was coded as a 1, if it was dedected in the open period, it was coded as 2. (DOC) [file pone.0137541.s002.doc]

| **Code** | **Month** | **Year** |
| --- | --- | --- |
| JM-12 | April | 2000 |
| JM-5 | April | 2000 |
| JF-10 | December | 2000 |
| JF-1 | February | 2000 |
| JM-3 | February | 2000 |
| JF-5 | May | 2000 |
| JM-3 | October | 2000 |
| JNI-8 | February | 2000 |
| JF-1 | December | 2001 |
| JF-8 | December | 2001 |
| JNI-1 | December | 2001 |
| JNI-7 | December | 2001 |
| JM-5 | January | 2001 |
| JF-6 | July | 2001 |
| JF-10 | June | 2001 |
| JNI-2 | March | 2001 |
| JM-9 | May | 2001 |
| JM-10 | December | 2002 |
| JM-6 | December | 2002 |
| JM-8 | March | 2002 |
| JF-1 | November | 2002 |
| JF-1 | October | 2002 |
| JM-10 | April | 2003 |
| JF-1 | March | 2003 |
| JF-4 | May | 2003 |
| JF-1 | May | 2003 |
| JF-10 | January | 2003 |
| JF-7 | May | 2004 |
